# Respiratory comorbidities and treatments in Duchenne muscular dystrophy: impact on life expectancy and causes of death

**DOI:** 10.1007/s00415-024-12372-7

**Published:** 2024-04-17

**Authors:** Lisa Wahlgren, Anna-Karin Kroksmark, Anders Lindblad, Mar Tulinius, Kalliopi Sofou

**Affiliations:** 1 The Queen Silvia Children’s Hospital, Sahlgrenska University Hospital, Gothenburg, Sweden; 2 Department of Pediatrics, Lnstitute of Clinical Sciences, Sahlgrenska Academy, University of Gothenburg, Gothenburg, Sweden; 3 Department for Health and Rehabilitation/Physiotherapy, University of Gothenburg, Gothenburg, Sweden

**Keywords:** Duchenne muscular dystrophy, Pulmonary, Survival, Cause of death, Risk factors

## Abstract

**Background:**

Duchenne muscular dystrophy (DMD) is a neuromuscular disorder with progressive decline of pulmonary function increasing the risk of early mortality. The aim of this study was to explore the respiratory-related comorbidities, and the effect of these comorbidities and treatments on life expectancy and causes of death.

**Methods:**

All male patients living in Sweden with DMD, born and deceased 1970–2019, were included. Data regarding causes of death were collected from the Cause of Death Registry and cross-checked with the medical records along with diagnostics and relevant clinical features.

**Results:**

Hundred and twenty nine patients were included with a median lifespan of 24.3 years. Acute respiratory failure accounted for 63.3% of respiratory-related causes of death. 70.1% suffered at least one pneumonia, with first episode at a median age of 17.8 years. Hypoventilation was found in 73.0% with onset at 18.1 years. 60.5% had their first pneumonia before established hypoventilation. Age at onset of hypoventilation showed a strong correlation with age at first pneumonia. First pneumonia and scoliosis non-treated with scoliosis surgery increased the risk of dying of respiratory-related causes. In 10% of the patients, first pneumonia resulted in acute tracheostomy or early death. Patients treated with assisted ventilation had higher life expectancy compared to untreated patients.

**Conclusions:**

Our results highlight the importance of identifying subclinical hypoventilation in a timely manner and the importance of an active treatment regime upon clinical signs of pneumonia.

## Introduction

Duchenne muscular dystrophy (DMD) is a severe X-linked neuromuscular disorder caused by mutations in the gene encoding for dystrophin, a protein expressed in the sarcolemma of the skeletal and cardiac muscles. Lack of dystrophin affects the myofibers, causing repetitive inflammation, necrosis and regeneration, making them more susceptible to contraction‐induced injury and muscle loss [1]. Affected boys typically show symptoms of DMD around 3–5 years of age, in form of proximal muscle weakness and pseudohypertrophy of the calves and are found to have increased levels of creatine kinase. Due to its progressive nature, the disease results in wheelchair confinement in in adolescence and death, usually from respiratory or cardiac failure, in early adulthood. Advances in respiratory care, glucocorticoid treatment and cardioprotection have substantially improved the overall survival in DMD [2, 3]. As a result, the average life expectancy has gradually increased to 30 years of age [4, 5].

Pulmonary function is a cardinal measure of disease severity in DMD [6]. The progressive weakness of the respiratory muscles accompanied by the inability to take deep breaths results in microatelectasis and restricted compliance of the lungs and chest wall [7, 8]. Restricted inspiration in combination with impaired expiratory muscle function leads to significant reductions in cough efficiency and mucociliary clearance [8]. This is particularly important in the event of respiratory tract infections, as secretion stagnation and subsequent hypoxia make patients with DMD prone to acute respiratory distress and recurrent pulmonary infections [8, 9]

Regular measurement of pulmonary function is the cornerstone of respiratory management in DMD, along with timely initiation of lung volume recruitment therapy (LVR), manually or mechanically assisted cough and assisted ventilation [10]. Lung volume recruitment therapy, i.e., by self-inflating ventilation, has positive effect on pulmonary function, and on peak cough flow in conjunction with manually assisted cough [11, 12]. Mechanical insufflation–exsufflation (MI-E), either alone or in combination with manual thrust, is considered the best treatment option to increase peak cough flow (PCF) in patients with ventilator-dependent neuromuscular disease [12]. The introduction of assisted ventilation in the 90s led to an increase of life expectancy in DMD from 19 to 30 years of age [13–15]. Patients with DMD treated with non-invasive mechanical ventilation in combination with manually assisted cough and cardioprotective medications were shown to have an increased life expectancy of approximately 40 years [2].

Pulmonary function is also affected by scoliosis and the secondary deformity of the ribcage. Scoliosis is still commonly occurring among patients with DMD, even if corticosteroid treatment appears to reduce the proportion of patients developing scoliosis [10, 16, 17]. Scoliosis commonly develops early after loss of ambulation, while rapid deterioration of scoliosis significantly correlates to increased pain, spinal rigidity, as well as impaired cardiac and pulmonary function [18, 19]. Patients with DMD and scoliosis of 20° or more should be evaluated by an orthopaedic surgeon, considering that scoliosis surgery is associated with improved pulmonary function and sitting balance [10, 20, 21].

The progressive decline of pulmonary function is a major cause of early mortality in DMD [22]. Despite the evolution of respiratory care, respiratory insufficiency still accounts for approximately 40% of primary causes of death [4]. There is a knowledge gap regarding the optimal initiation time for LVR and MI-E [11, 23, 24]. Moreover, there is limited knowledge regarding the impact of respiratory diseases and related comorbidities, and interventions, such as scoliosis surgery, on mortality and causes of death in DMD. Our aim with this study was to explore the respiratory-related comorbidities in patients with DMD, and the potential effect of these comorbidities and associated treatments on life expectancy and causes of death.

## Materials and methods

### Study population

All male patients living in Sweden with a confirmed diagnosis of DMD, born since 1970 and deceased by the end of 2019, were included in the study. As this was a mortality and cause-of-death-based study only deceased patients were included. Patients’ life expectancy and causes of death and the methodology applied have been described in detail elsewhere [4]. Patients were identified by multiple data sources, i.e., pathology laboratories for skeletal muscle biopsies, registries [the National Quality Registry for Neuromuscular Diseases in Sweden (NMiS) and the Swedish Registry of Respiratory Failure (Swedevox)], neurology and respiratory clinics and the national network of clinicians working with neuromuscular diseases in Sweden. The Cause of Death Registry at the Swedish National Board of Health and Welfare was used to identified deceased patients. The inclusion criteria applied were (1) a typical clinical phenotype of DMD with elevated serum creatine kinase AND (2) either (i) muscle biopsy findings compatible with DMD or (ii) pathogenic DMD variants in the dystrophin gene or (iii) confirmed DMD diagnosis in a maternal relative. A typical clinical phenotype was defined as symptom onset before 5 years of age and loss of ambulation before 13 years of age [25], or before 16 years of age for patients receiving corticosteroid treatment [26].

### Data collection

Data regarding the causes of death were collected from the Cause of Death Registry at the Swedish National Board of Health and Welfare. Patients’ medical records were used to cross-check data on causes of death, as well as to collect data on diagnostics and clinical features, including age at loss of ambulation (LoA), comorbidities and treatments. Data were systematically collected with the use of a case report form.

### Definitions of variables

Age at onset of hypoventilation was defined as the patient age when the need of assisted ventilation was first documented in the medical records. First pneumonia was considered the first episode of pneumonia that was diagnosed either in a hospital setting or by a specialist in primary care. Significant scoliosis, referred from this point forward as scoliosis, was defined as scoliosis with a cobb angle over 20° or described in the medical records as “clinically visible”, “clear signs of”, or “pronounced”. Death by acute respiratory failure was considered when the leading cause of death was pneumonia, pneumothorax, secretion stagnation or in the case of sudden death at home, directly connected to ventilator malfunction.

Treatment with glucocorticoids was defined as treatment lasting more than 6 months, while those patients treated for less than 6 months were considered treatment-naïve. Treatment with assisted ventilation was categorized in non-invasive ventilation and invasive ventilation (i.e., tracheostomy), and was further described when exceeding 16 h per day. Treatment with MI-E was considered to be ongoing after being prescribed and introduced to the patient, despite the level of use. Cardiomyopathy was considered upon abnormal echocardiography findings [i.e., ejection fraction (EF) less or equal to 44% or shortening fraction (SF) less or equal to 19%] or, clinically, when described in the medical records as “moderate”, “severe”, or “pronounced” [27, 28].

### Statistical analysis

Descriptive data is presented as median and interquartile range for numeric variables. Categorical variables are presented in numbers and percentages. Wilcoxon rank sum test and Chi-square test were used for comparison between groups. Correlation analyses between age at first pneumonia, age at onset of hypoventilation, age at LoA and age at scoliosis > 20° were performed using Spearman rank correlation coefficients, with the strength and direction of r reported together with an interpretation of the strength [29]. Time to event outcomes were analyzed using time-to-event analysis. Kaplan–Meier curves and log rank test were used for comparisons between groups. Cumulative incidence functions were used to describe the cumulative incidence of events over time, accounting for competing events. Risk factors for death by respiratory causes were analyzed using cause-specific Cox proportional hazards regression with censoring for competing events, i.e., with death by other cause as competing risk for death by respiratory causes. The Cox regression analyses were adjusted for the potential confounding factor ‘year of birth’ (1970–1979, 1980–1989, and 1990 or later). Results are presented as hazards ratios (HRs) with 95% confidence intervals (CIs). All significance tests were two-sided and conducted at the 5% significance level. Statistical analyses were performed using SAS/STAT^®^ Software, Version 9.4 of the SAS System for Windows (SAS Institute, Cary, N.C.) and IBM^®^ SPSS^®^ statistics, version 28.0.1.1 (© Copyright IBM Corporation 1994, 2023).

## Results

### Study population

**Fig. 1:**
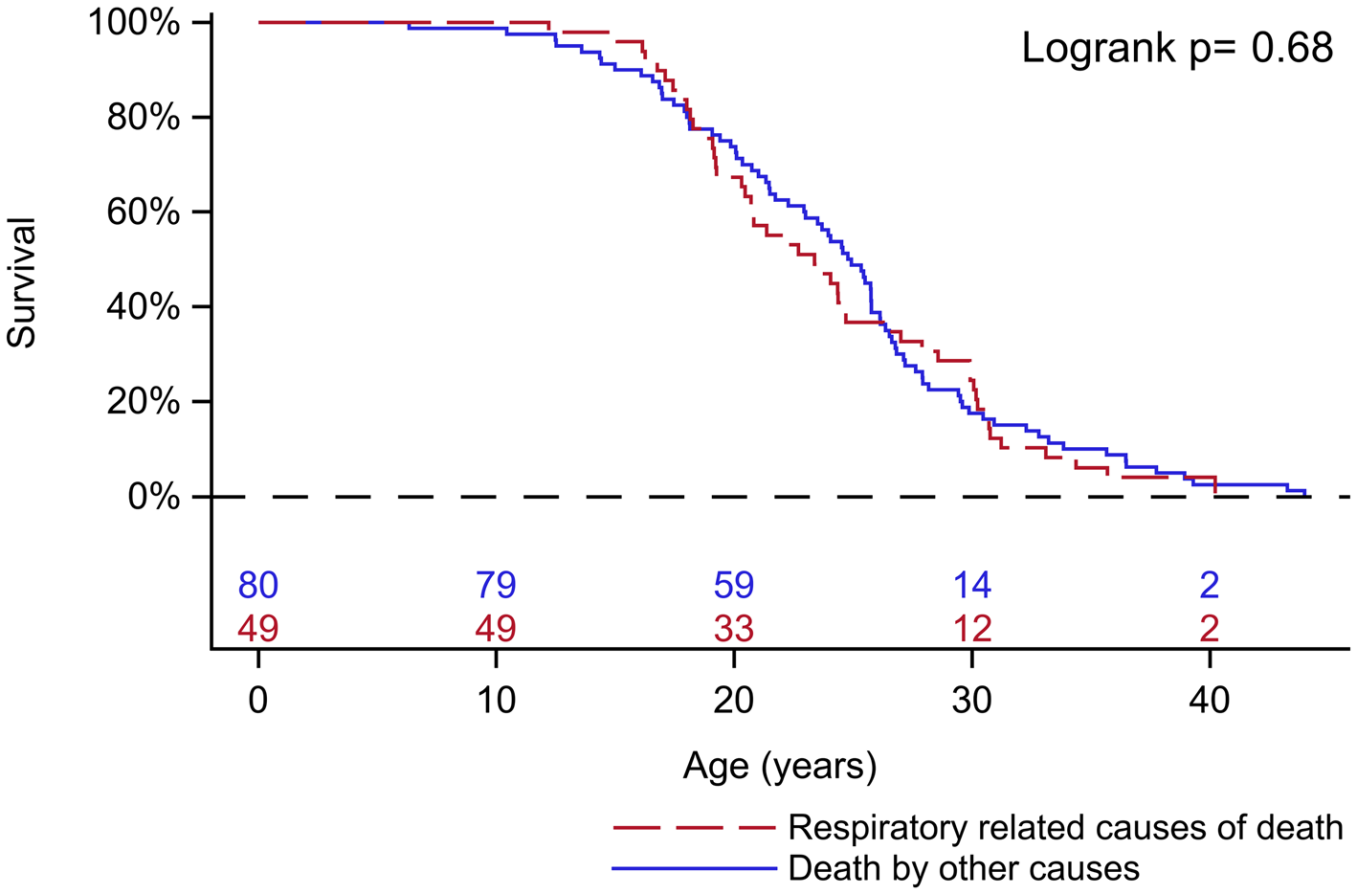
Kaplan–Meier survival curve for respiratory and other causes of death (*n* = 129)

In total, 129 patients were identified and included in this study, of whom 45 (35%) had a genetic diagnosis. The median (IQR) age of death was 24.3 years (19.1–29.0). The most common leading cause of death was heart complications (*n* = 54, 41.9%), followed by respiratory-related causes (*n* = 49, 38.0%) and non-cardiopulmonary causes (*n* = 26, 20.1%) (Table 1). The median (IQR) age of death due to respiratory-related causes was 23.4 (18.9–30.0) years. Acute respiratory failure accounted for 63.3% of respiratory-related causes of death at a median (IQR) age of 21.4 years (19.2–27.9). There was no age difference between patients who died of respiratory-related causes compared to other causes of death (log rank *p = *0.68; Fig. 1). Demographics and clinical events for the total study population and for the subgroups of patients born in the 80 s and the 90 s are presented in Table 1.

**Table 1:** Demographics and clinical events for the total study population (*n* = 129) and for the subgroups of (a) patients born in the 80 s (*n* = 63) and (b) patients born in the 90 s (*n* = 37)

Total population		By decade of birth	
	1970–2019	1980–1989	1990–1999
Deceased patients	*n* = 129	*n* = 63	*n* = 37
Median (IQR) lifespan, years	24.3 (19.1–29.0)	25.5 (20.7–28.2)	19.9 (14.4–22.5)
Glucocorticoids, n (%)	68 (59.6%)	31 (56.4%)	29 (90.6%)
Median age at start (IQR) years	6.5 (5.0–8.5)	7.5 (5.8–9.2)	5.4 (4.0–6.5)
Loss of ambulation, n	127	61	37
Median (IQR) age, years	10.0 (9.0–11.9)	10.0 (8.5–11.0)	11.0 (9.2–12.0)
Scoliosis, n (%)	95 (79.8%)	51 (86.4%)	23 (67.6%)
Median (IQR) age, years	14.4 (12.3–15.6)	14.4 (12.3–15.6)	14.6 (11.9–15.5)
Scoliosis surgery n (%)	46 (48.4%)	26 (51.0%)	17 (73.9%)
Median (IQR) age, years	15.4 (14.1–17.3)	15.1 (14.2–16.1)	15.8 (14.1–18.2)
Mechanical in-exsufflation, n (%)	53 (43.8%)	32 (54.2%)	16 (47.1%)
Median (IQR) age, years	20.3 (17.4–25.4)	23.4 (19.0–26.0)	17.4 (14.6–19.8)
Pneumonia, *n* (%)	82 (70.1%)	45 (77.6%)	22 (66.7%)
Median (IQR) age (first), years	17.8 (13.7–22.4)	17.2 (14.2–23.2)	14.9 (12.1–20.6)
Hypoventilation, *n* (%)	92 (73.0%)	49 (80.3%)	20 (54.1%)
Median (IQR) age, years	18.1 (15.6–20.8)	18.1 (16.7–20.6)	15.3 (13.7–18.5)
Assisted ventilation, *n* (%)	89 (71.2%)	47 (77.0%)	19 (52.8%)
Median (IQR) age, years	19.3 (17.0–22.0)	19.3 (17.4–22.3)	18.3 (15.2–19.7)
Tracheostomy, *n* (%)	23 (18.3%)	14 (23.0%)	3 (8.1%)
Median (IQR) age, years	24.9 (19.0–30.5)	25.5 (19.2–29.1)	18.2 (14.4–)
Causes of death			
Respiratory, *n* (%)	49 (38.0%)	28 (44.4%)	12 (32.4%)
Cardiac, *n* (%)	54 (41.9%)	29 (46.0%)	12 (32.4%)
Non-cardiopulmonary, *n* (%)	26 (20.1)	6 (9.5%)	13 (35.1%)

*N* number, *IQR* inter quartile range, presented as range from 75th percentile to 25th percentile

Missing data where applicable: Treated with corticosteroids 15/129; age at start of glucocorticoids 5/68; scoliosis 10/129; age at scoliosis 10/95; scoliosis surgery 10/129; Mechanical in-exsufflation 8/129; pneumonia 12/129; age at first pneumonia 7/82; hypoventilation 3/129; age at confirmed hypoventilation 2/92; assisted ventilation 4/129; age at start of assisted ventilation 2/89; tracheostomy 3/129

### Clinical events

Eighty-two (70.1%) of 117 patients suffered at least one episode of pneumonia, while 45 (54.9%) of these patients had two or more episodes of pneumonia during their lifetime. Forty-six (60.5%) of 76 patients had their first pneumonia before they had an established hypoventilation. Of the 92 patients with hypoventilation, 37 (40.2%) died of respiratory-related causes and 42 (45.7%) died of cardiac complications (Table 1).

**Fig. 2:**
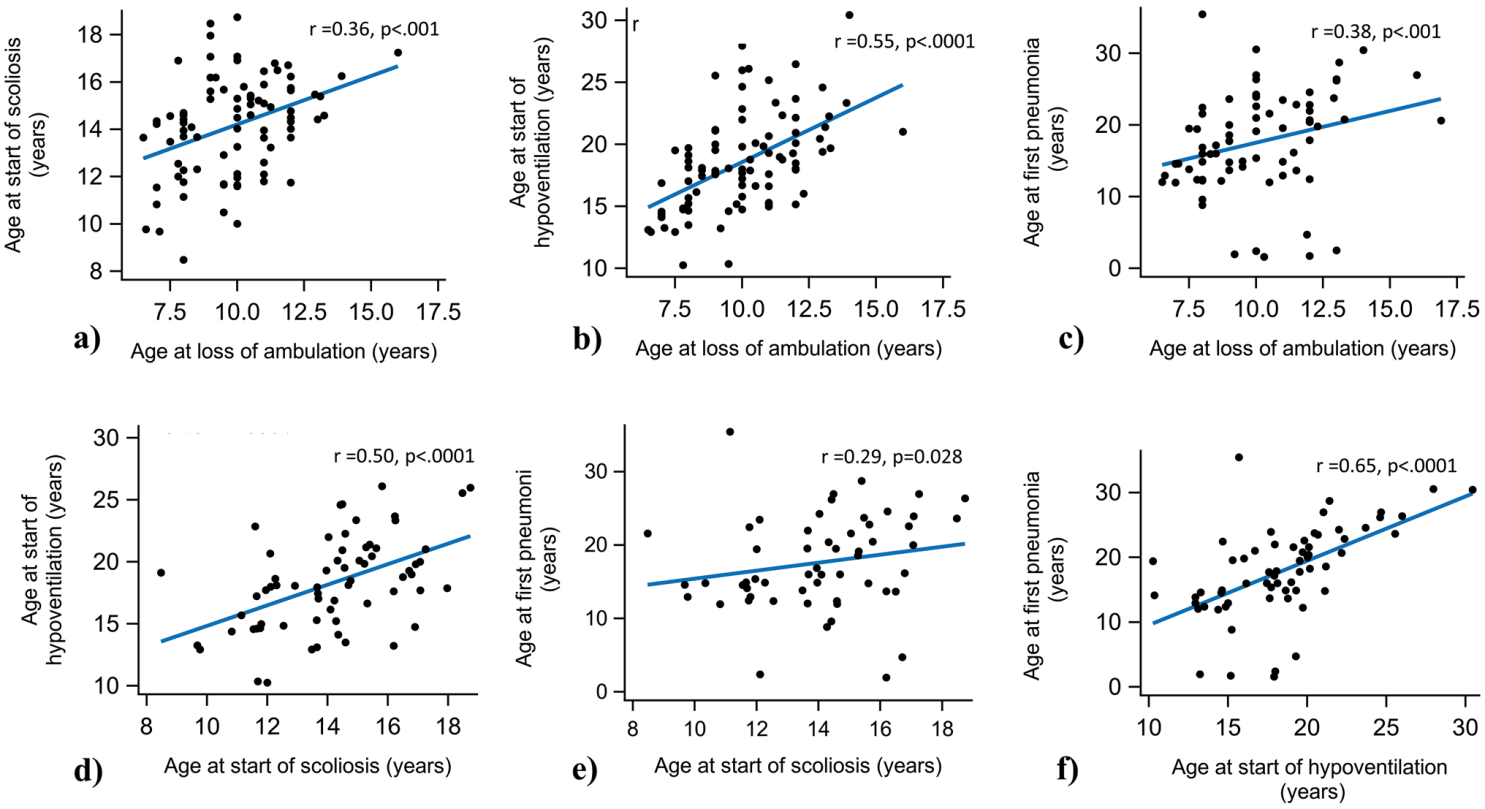
Spearman’s correlation analysis for clinical events: **a** age at loss of ambulation (LoA) and age at scoliosis (*n* = 85); **b** age at LoA and age at start of hypoventilation (*n* = 90); **c** age at LoA and age at first pneumonia (*n* = 75); **d** age at scoliosis and age at start hypoventilation (*n* = 65); **e** age at scoliosis and age at first pneumonia (*n* = 56); and **f** age at start of hypoventilation and age at first pneumonia (*n* = 63)

The occurrence of pneumonia was slightly less frequent among patients born in the 90 s compared to the 80 s (Table 1). There was no difference in the occurrence of pneumonia before established hypoventilation between these two groups (63.6% vs 61.9% of patients). Age at onset of hypoventilation showed a strong correlation with age at first pneumonia (r = 0.65, *p = * < 0.001) and a fair-to-moderate correlation with age at LoA (r = 0.55, *p = * < 0.001) and age at scoliosis (r = 0.50, *p = * < 0.001). The correlations between these clinical events, i.e., age at LoA, scoliosis, first pneumonia and onset of hypoventilation, are depicted in Fig. 2a–f.

### Respiratory-related interventions

Treatment with glucocorticoids was initiated in 68 (59.6%) of 114 and 32 (47.1%) of these patients stopped prematurely at a median (IQR) age of 11.0 (10.3–14.6) years (*n* = 31). Forty-six (48.4%) of 95 patients with scoliosis underwent scoliosis surgery. The amount of patients treated with glucocorticoids and scoliosis surgery were increasing with each decade (Table 1).

**Fig. 3:**
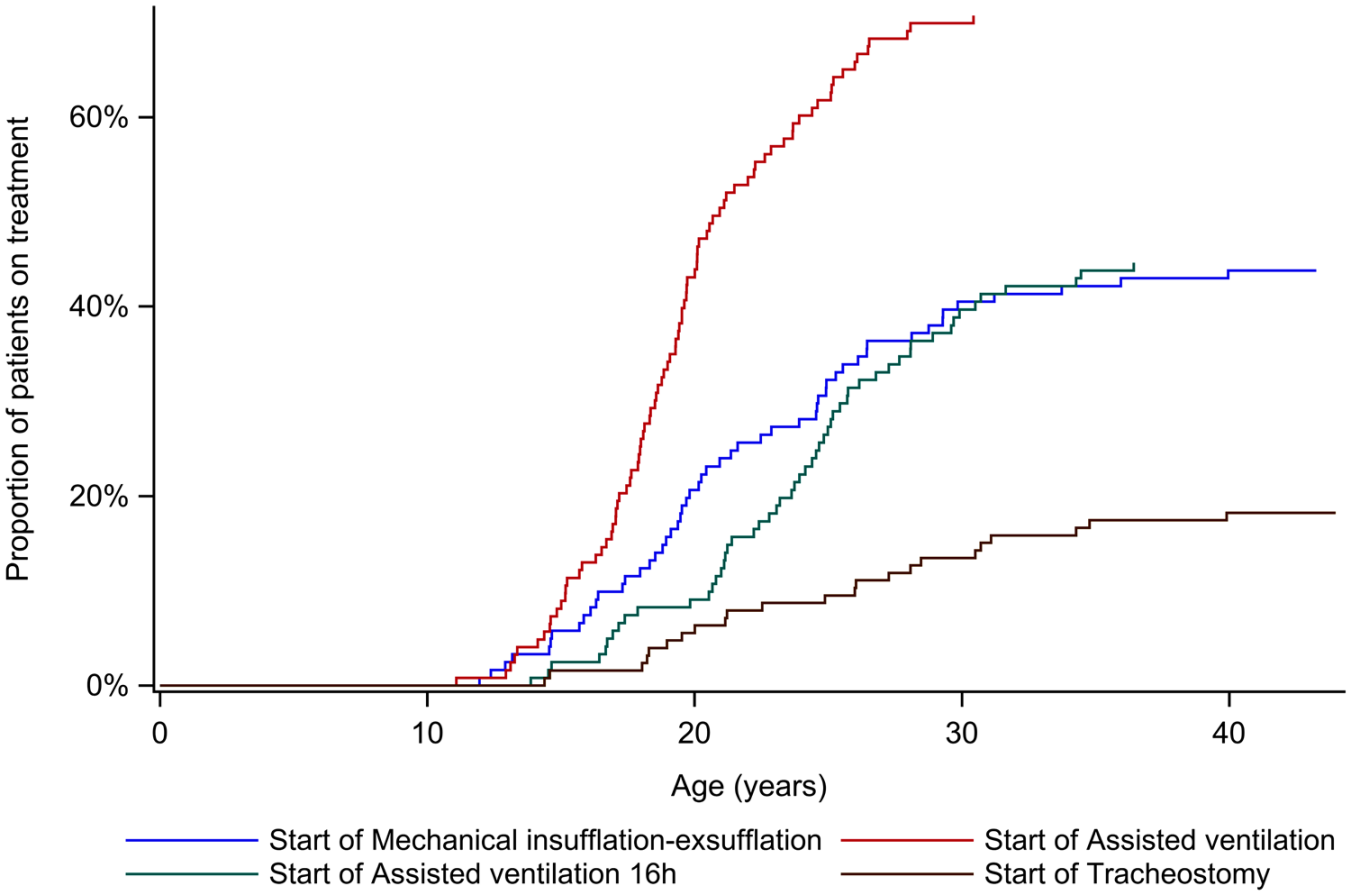
Age at start of treatment with Mechanical insufflation–exsufflation (MI-E) (*n* = 53), assisted ventilation (*n* = 87), assisted ventilation more than 16 h per day (*n* = 54) and tracheostomy (*n* = 23)

Assisted ventilation was initiated in 89 (71.2%) of 125 patients and 57 (46.0%) of 124 patients were treated with assisted ventilation more than 16 h per day. Age at respiratory-related interventions, including MI-E and assisted ventilation, is presented in Fig. 3. Median (IQR) time from start of assisted ventilation to treatment more than 16 h was 4.1 (2.0–7.4) years (*n* = 52). The age at MI-E initiation decreased over the years (Table 1). Twenty-three (18.3%) of 126 patients received tracheostomy at a median (IQR) age of 24.9 (19.0–30.5) years. Acute tracheostomy was more common than elective; 15 (65.2%) of 23 patients underwent acute tracheostomy at a median (IQR) age of 26.0 (19.0–30.7) years and for 5 of them, tracheostomy was performed during their first pneumonia. Six patients underwent elective tracheostomy, at a median (IQR) age of 24.3 (20.5–29.2) years. For two patients, the reason for tracheostomy was unknown.

### Impact of clinical events and respiratory-related interventions on life expectancy

Scoliosis surgery had no significant effect on life expectancy. The median (IQR) age of death for those who underwent scoliosis surgery was 24.4 (20.6–28.0) years (*n* = 46), compared to those patients with scoliosis who did not undergo surgery, who died at 25.4 (18.6–29.9) years (*n* = 49); *p = *0.82. There was no significant difference in median (IQR) age at start of hypoventilation between the patients who underwent scoliosis surgery compared to those who did not undergo surgery [18.4 (15.3–20.9) years (*n* = 34) vs 18.0 (15.2–20.7) years (*n* = 40); *p = *0.67]. Developing significant scoliosis and not being treated with scoliosis surgery increased the risk of dying in respiratory-related causes of death [HR 2.66 (95% CI 1.01–7.01); *p = *0.047].

**Fig. 4:**
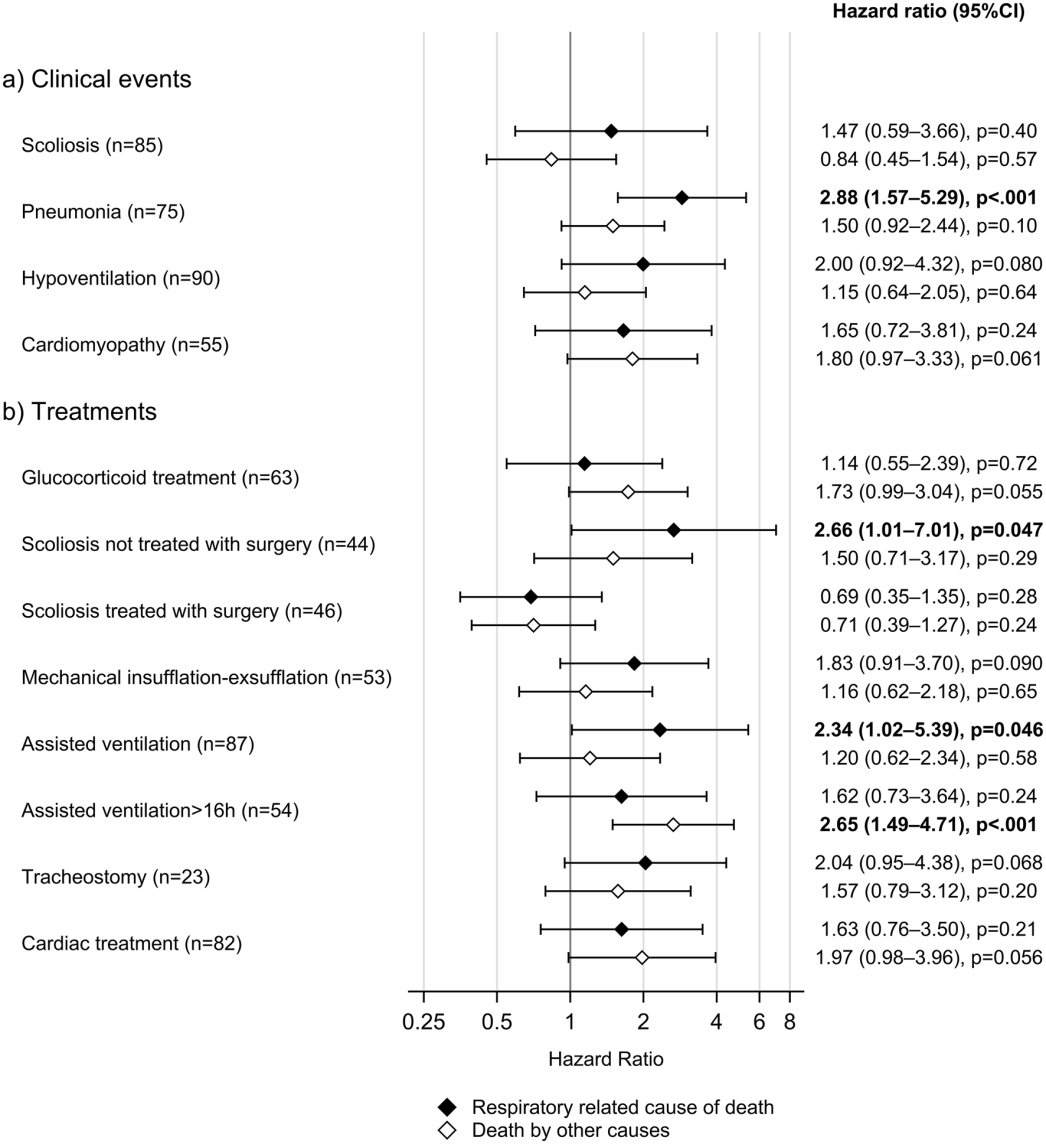
Risk factors for death by respiratory-related causes and by other causes, devided by a) clinical events and b) treatments. Number of patients in parentheses. Analyses with cause-specific Cox proportional hazards regression with censoring for death by other cause as competing risk for death by respiratory causes. The Cox regression analyses were adjusted for potential confounding from year of birth (1970–1979, 1980–1989, and 1990 or later). *CI* confidence intervals, number of patients in parentheses

Acute respiratory infection was the leading cause of death in 14% of the total population and 37% of those who died in respiratory-related causes. A history of pneumonia was significantly more common in patients who died of respiratory-related causes compared to other causes of death (80.4% vs 63.4%; *p = *0.049) (Table 2). The risk of dying of respiratory-related causes increased significantly after the first pneumonia [HR 2.88 (95% CI 1.57–5.29); *p = * < 0.001] (Fig. 4). The first episode of pneumonia was also the leading cause of death in four patients. None of these four patients had established heart failure, while two were treated with corticosteroids even after LoA. The median age of death for those four patients was 19.8 years of age, the youngest being in his early teenage years and the oldest close to 21 years of age. Pneumonia was the reason for unplanned hospitalization during the last year of life for 24 patients, of whom nine died of respiratory-related causes.

**Table 2:** Clinical events in patients who died of respiratory vs other causes of death

	Patients who died of respiratory-related causes of death (*n* = 49)	Missing, *n* (%)	Patients who died of other causes of death (*n* = 80)	Missing, *n* (%)	*p* value
Loss of ambulation, *n* (%)	49 (100%)	0 (0%)	80 (100%)	0 (0%)	na
Median age at Loss of Ambulation (IQR), years	10.0 (8.0–11.0)	0 (0%)	10.3 (9.0–12.0)	0 (0%)	**0.023**
Scoliosis, *n* (%)	41 (89.1%)	3 (6.1%)	54 (74.0%)	7 (8.8%)	**0.045**
Median age at scoliosis (IQR), years	14.4 (12.0–15.6)	7 (17%)	14.3 (12.6–15.6)	3 (5.6%)	0.95
Pneumonia, *n* (%)	37 (80.4%)	3 (6.1%)	45 (63.4%)	9 (11.3%)	**0.049**
Median age at first pneumonia (IQR), years	17.2 (12.9–22.4)	2 (5.4%)	18.2 (13.9–22.5)	5 (11.1%)	0.54
Mechanical in-exsufflation (MI-E), *n* (%)	22 (45.8%)	1 (2.0%)	31 (42.5)	7 (8.8%)	0.72
Median age at start MI-E (IQR), years	21.2 (16.3–25.7)	0 (0%)	20.1 (18.3–24.9)	0 (0%)	0.99
Hypoventilation, *n* (%)	37 (77.1%)	1 (2.0%)	53 (68.8)	3 (3.8%)	0.32
Median age hypoventilation (IQR), years	17.7 (15.1–20.5)	0 (0%)	18.8 (16.7–21.0)	0 (0%)	0.21
Mechanical ventilation > 16 h, *n* (%)	20 (42.6%)	2 (4.1%)	37 (48.1%)	3 (3.8%)	0.55
Median age Mechanical vent > 16 h (IQR), years	23.2 (18.6–28.9)	3 (15.0%)	24.4 (21.1–27.1)	0 (0%)	0.58
Tracheostomy, *n* (%)	10 (20.8%)	1 (2.0%)	13 (16.7%)	2 (2.5%)	0.56
Median age tracheostomy (IQR), years	22.8 (18.3–32.0)	0 (0%)	24.9 (20.6–29.3)	0 (0%)	0.98

Categorical variables: Chi-square; *p = * < 0.05

Continuous variables: Wilcoxon rank sum test; *p = * < 0.05

*n* number, *y* years, *IQR* inter quartile range, presented as range from 75th percentile to 25th percentile, *h* hours

Patients treated with assisted ventilation (invasive and/or non-invasive) had significantly higher life expectancy; the median (IQR) age of death was 26.1 years (*n* = 89; 95% CI 24.8–27.4), compared to untreated patients who died at a median age of 18.0 years (*n* = 36, 95% CI 17.7–18.3; log rank *p = * < 0.001). Additional treatment with MI-E did not have significant impact on life expectancy; the median (IQR) age of death was 27.0 years (*n* = 49, 95% CI 25.8–28.3) compared to patients treated with assisted ventilation but not MI-E who died at a median (IQR) age of 25.3 years (*n* = 40, 95% CI 25.8–28.3; log rank *p = *0.77). Patients treated with non-invasive ventilation died at median (IQR) age of 25.6 (21.4–29.5) years (*n* = 66). Patients treated with tracheostomy died at a median age of 30.1 (24.8–33.8) years (*n* = 23).

Requiring assisted ventilation (invasive and/or non-invasive) significantly increased the risk of dying from respiratory-related causes [HR 2.34 (95% CI 1.02–5.39); *p = *0.046]. Requiring assisted ventilation greater than 16 h per day was not associated with a significant risk of dying from respiratory-related causes [HR 1.62 (95% CI 0.73–3.64); *p = *0.24].

## Discussion

We identified two clinical variables that significantly increased the risk of dying from respiratory-related causes in DMD: (a) the first episode of pneumonia and (b) the development of significant scoliosis non-treated with scoliosis surgery. Acute respiratory failure including pneumonia accounted for 63.3% of respiratory-related causes of death, pneumonia being a major cause of unplanned hospital admission during the last year of life. The first episode of pneumonia, typically occurring before adulthood and for the majority before established hypoventilation, was accompanied by severe clinical outcomes in 10% of the patients, including acute tracheostomy and early death. Finally, we showed that treatment with assisted ventilation appears to have a significant impact on life expectancy.

Patients with neuromuscular disorders have a well-known risk for acute pulmonary infections, mainly due to progressive respiratory muscle loss and weakened coughing [8, 9]. The most common trigger is upper respiratory tract infections [30]. At later stages, colonization may occur by pathogens such as Pseudomonas aeruginosa, methicillin resistant Staphylococcus aureus (MRSA) and other multidrug-resistant microorganisms [31]. In this study, the first episode of pneumonia commonly occurred at late teen years, as seen in previous research [32], and often prior to the initiation of assisted ventilation. This does not exclude the possibility of undiagnosed hypoventilation or intermittent hypoxia secondary to sleep disordered breathing; conditions that would make patients susceptible to community-acquired pneumonias [30, 33]. Although not systematically assessed in this study, dysphagia-induced aspiration might be another underlying cause of acute pulmonary infections. In DMD, dysphagia has been associated with higher risk for aspiration pneumonia, the risk increasing with advancing age [34]. The supraglottic aspiration and accumulation of residue seen in patients with dysphagia may lead to silent aspiration, which is presumed to be a common cause of aspiration pneumonia and sudden death among non-ambulatory patients with DMD [35]. We found a strong correlation between the age at first pneumonia and the age at onset of hypoventilation. Our findings suggest that there was either a pre-existing hypoventilation that went on unnoticed or that pneumonia induced a respiratory failure.

The forced vital capacity (FVC) in DMD is known to begin its decline from the age of 10–12 years, at an annual rate of 5% [36]. Drop of FVC below 1 L is considered a marker for increased mortality [22]. We found that treatment with assisted ventilation, invasive or not, was associated with significantly higher life expectancy, thus confirming previous research [3, 13, 14]. Close monitoring for signs of hypoventilation is, therefore, crucial in DMD to ensure timely initiation of ventilatory support. Moreover, initiation of LVR treatment is recommended when forced vital capacity (FVC) is below 60% of the predicted value [10]. Recent research postulates that assisted cough (manual or mechanical) should precede LVR, as expiratory muscles are severely affected before FVC falls below 60% of the predicted value [37]. In this study, the majority of the patients received mechanically assisted cough after initiation of assisted ventilation. The late introduction of assisted cough might also contribute to the increased occurrence of pneumonia prior to ventilatory support.

Progressive scoliosis in conjunction with respiratory muscle weakness have a negative impact on pulmonary function [10, 16]. Scoliosis surgery has been shown to improve the rate of decline in pulmonary function in DMD [20, 38, 39], but not the frequency of chest infections [38]. Studies investigating the impact of scoliosis surgery on life expectancy have shown contradictory results, presumably owing to baseline differences in the Cobb angle and spinal deformities, functional status and FVC between the surgical and non-surgical groups [39]. To our knowledge, mortality and causes of death in surgically treated vs non-treated patients with DMD have not been previously assessed. We found that non-surgically treated patients with significant scoliosis are at increased risk of dying from respiratory-related causes. Our results point out the importance of a combined monitoring of scoliosis and pulmonary function in DMD and the urgency of having respiratory-specific protocols in place to promptly apply in progressive scoliosis, especially when surgery is delayed or contraindicated.

Strengths of this study are its unique nationwide cohort of 129 deceased patients with DMD and that data were collected from medical records in a structured, double-review manner. Due to its retrospective, cause-of-death based methodology, this study has some limitations worth noting. Patients still alive by the end of 2019 were not included, limiting the statistical methods applied. To address the evolution of disease management throughout the decades and minimize the risk of skewed data, we considered the year of birth as a potential confounding factor; therefore, regression analyses were adjusted for that. Missing data ranged from 0 to 17%, with the exception for cardiomyopathy assessment during the last year of life with 31% missing data. To ensure high quality of collected data, we excluded those data that could not be confirmed, i.e., in the case of a treatment that was either not given, or there was missing documentation in the medical record, it was considered as missing data. The patients’ compliance to initiated treatments, when not documented, could not be assessed and, therefore, not included in data analysis.

Patients with neuromuscular diseases may present poor adherence to preventive measures and treatment protocols [40], and this is not an exception for patients with DMD, especially during the early stages of the disease. We found the occurrence of pneumonia in DMD to carry a high risk of severe clinical outcomes including early death. We also found that the onset of hypoventilation was strongly associated with first pneumonia. Our results highlight the importance of identifying subclinical hypoventilation in a timely manner. As dysphagia and gut dysmotility commonly develop in non-ambulatory patients [41], regular assessment of swallowing function should be considered in non-ambulatory patients with DMD. In most cases of pneumonia the causative pathogen is unknown at first and the use of empiric antibiotics should be considered early upon clinical signs of pneumonia [10, 42]. Applying respiratory-specific protocols has been shown to lower the risk of hospitalization due to severe chest infections and demonstrated the importance of coughing aids in acute situations [43]. The incorporation of respiratory-specific protocols in the management of patients with DMD should also include guidelines for infection management and timelines for the introduction of respiratory aids. Other factors to consider is ways to enhance adherence in early stages, i.e., before established hypoventilation, as well as to harmonize the monitored variables to ensure that patients receive optimal management depending on the stage of disease.

## Data Availability

The data that support the findings of this study are available from the corresponding author upon reasonable request.
